# Women’s Empowerment in India: State-Wise Insights From the National Family Health Survey 5

**DOI:** 10.7759/cureus.65509

**Published:** 2024-07-27

**Authors:** B Vignitha, Aninda Debnath, Aditya PVS, Teja K Sai, Shweta Charag

**Affiliations:** 1 Community Medicine, Vardhman Mahavir Medical College and Safdarjung Hospital, New Delhi, IND; 2 Epidemiology, Sun Yat-sen University, Guangzhou, CHN; 3 Epidemiology, Odessa National Medical University, Odessa, UKR

**Keywords:** socio-economic factors, state-wise analysis, india, nfhs-5, women's empowerment

## Abstract

Background

Women’s empowerment is critical for achieving gender equality and societal progress. Despite various efforts, significant disparities in women’s empowerment persist across different states in India. This study aims to provide a comprehensive assessment of women’s empowerment using data from the National Family Health Survey 5 (NFHS-5).

Methods

Data from the NFHS-5, collected between June 2019 and April 2021, were used. The Women’s Empowerment Index (WEI) was calculated for each of the 28 states based on four dimensions: economic empowerment, decision-making, health and nutrition, and gender roles. Statistical analysis, including Pearson and Spearman correlations, was conducted to assess associations between WEI and various socioeconomic variables.

Results

The WEI ranged from 17.4 to 27.4, with a mean of 21.3 ± 2.6. Goa, Sikkim, and Himachal Pradesh had the highest WEI scores, while West Bengal, Andhra Pradesh, and Telangana had the lowest. Economic empowerment was highest in Karnataka, Sikkim, and Arunachal Pradesh. Decision-making scores were highest in Nagaland, Mizoram, and Goa. Health and nutrition scores were highest in Goa, Sikkim, and Uttarakhand. Positive gender roles were most prominent in Himachal Pradesh, Nagaland, and Goa. Significant correlations were found between WEI and per capita net state domestic product, literacy rates, median age at marriage, and total fertility rate.

Conclusion

The study highlights substantial variations in women’s empowerment across Indian states, influenced by socioeconomic, health, and educational factors. Targeted interventions are needed to address specific barriers and promote gender equality. Future research should evaluate the effectiveness of these interventions and explore additional factors influencing women’s empowerment.

## Introduction

To empower is to strengthen. To empower is to gain control over oneself. To empower is to attain equality. To empower is to move forward as an individual and as a society. In an era where well-being is not the only goal, the equity approach aims at the upliftment of all, leaving behind none. However, statistically, women are more disadvantaged and, hence, more powerless. The ideology of empowerment is particularly pertinent to the powerless, making “women’s empowerment" a critical need in a still gender-discriminated society [1].

An empowered individual possesses greater intrinsic abilities and can thus overcome extrinsic challenges. This sense of empowerment revolves around self-awareness and self-confidence, which are powered through health, wealth, education, familial roles, decision-making, and overall well-being [2]. Despite these ideals, the literacy rate among women is only 64.6%, compared to 80.9% among men [3]. Furthermore, only 22% of women above the age of 15 are employed, in stark contrast to the 71% employment rate of men in the same age group [4].

This disparity is rooted in a lack of opportunities due to the neglect of rights by parents and guardians, child marriage, sexual and domestic violence, adolescent pregnancies, and poverty. The United Nations Population Fund suggests that “motherhood during childhood” disrupts education, job access, and progress beyond the poverty line, thereby exposing young mothers to related health hazards [5,6]. Nationally, politically, women represent only 13.4% of the total membership of parliament [7]. This underrepresentation at key levels of national development clearly indicates the systemic disempowerment of women. The “glass ceiling” effect, referring to invisible barriers preventing women from advancing to leadership positions, contributes significantly. Despite their qualifications, women face institutional biases, cultural norms, and discriminatory practices that limit their political opportunities. This effect hinders gender equality in representation and perpetuates disempowerment [8].

According to the Global Gender Gap Index 2021, India is the third worst country in South Asia, coming in at position 140 out of 156 [9]. This ranking underscores the deep-rooted patriarchy, traditions, systems, and beliefs that continue to oppress women. The demolition of such practices necessitates the elevation of girls’ value in society.

Given these persistent disparities and challenges, it is crucial to analyze and understand the factors that contribute to women’s empowerment in India. This study aims to provide a comprehensive assessment of women’s empowerment across various states using data from the National Family Health Survey (NFHS) [10]. By identifying key indicators and correlating them with socioeconomic variables, this research seeks to inform policies and interventions that can effectively promote gender equality and empower women in India.

## Materials and methods

The NFHS 5, a Demographic and Health Surveys (DHS) that was conducted in two phases (phase one from June 17, 2019, to January 30, 2020, and phase two from January 2, 2020, to April 30, 2021), provided the data used in this study. Seventy-two field agencies collected the data, which included information from 101,839 men and 724,115 women living in 636,699 homes. Men and women between the ages of 15 and 49 were included in the study, with a five-year spouse age gap taken into account. The sample size for India as a whole is roughly 610,000 homes [10].

Four survey schedules (household, woman’s, man’s, and biomarker) were conducted utilizing computer-assisted personal interviewing (CAPI) in the local languages. Information about women’s marriage, fertility, contraception, children’s healthcare and vaccinations, nutrition, sexual behavior, reproductive health, women’s empowerment, and domestic abuse was gathered for the women’s schedule.

**Figure 1:**
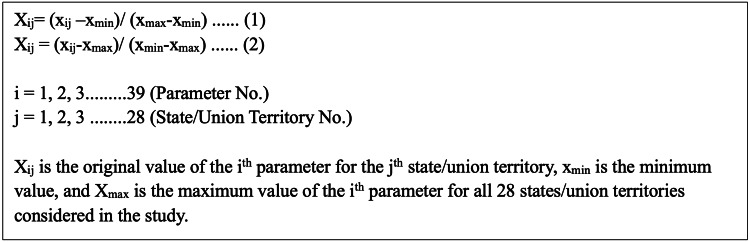
Standardization of parameters

The Women’s Empowerment Index (WEI) was calculated based on four dimensions: economic empowerment, decision-making, health and nutrition, and gender roles, each comprising various indicators and parameters, totaling 39 parameters as shown in Table 1. Economic empowerment included women’s employment status, access to money, credit, freedom of movement, and asset ownership. Decision-making covered women’s control over cash earnings and participation in household decisions. Health and nutrition encompassed knowledge and use of contraceptive methods and nutritional status. Gender roles focused on gender role attitudes and experiences of violence. To ensure comparability, the parameters were standardized. The WEI was then computed by summing these standardized parameters across the four dimensions, providing a comprehensive measure of women’s empowerment (Figure 1). The highest score for WEI was 39, and the lowest score was 0.

**Table 1:** Representation of the dimensions, indicators, and parameters used to construct the WEI IUD, intrauterine device; PPIUCD, postpartum intrauterine contraceptive device; WEI, Women’s Empowerment Index

Dimensions	Indicators	Parameters
Economic empowerment	Employment status of women	Percentage of women who are not employed in the 12 months preceding the survey
	Women's access to money, credit, and freedom of movement	Percentage of women who have money that they can decide how to use
		Percentage of women who have bank or savings accounts that they themselves can use
		Percentage of women who are allowed to go to three specified places alone
		Percentage of women who have taken a loan from a microcredit program
		Percentage of women who use mobile phones for financial transactions
	Ownership of assets by women	Percentage of women who own a house alone or jointly
		Percentage of women who own land alone or jointly
		Percentage of women who have a mobile phone that they themselves use
Decision-making	Control over the cash earnings of women	Alone or jointly with their husband decide how their own cash earnings are used
		Alone or jointly with their husband decide how their husband’s cash earnings are used
		Earn more or about the same as their husband
	Participation of women in decision-making	Own health care
		Making major household purchases
		Visits to her family or relatives
Health and nutrition	Knowledge of contraceptive methods	Percentage of women who know female sterilization
		Percentage of women who know about pills
		Percentage of women who know about condoms/Nirodh
		Percentage of women who know about PPIUCD/IUD
	Use of contraceptive methods	Percentage of women who use female sterilization
		Percentage of women who use pills
		Percentage of women who use condoms/Nirodh
		Percentage of women who use PPIUCD/IUD
	Nutritional status of women	Percentage of women with chronic energy malnutrition
		Percentage of women having any anemia (<12 g/dl)
		Percentage of women covered by a health scheme or insurance
	Women’s food consumption	Percentage of women consuming milk or curd
		Percentage of women consuming dark green, leafy vegetables
		Percentage of women consuming fish
		Percentage of women consuming chicken or meat
		Percentage of women consuming pulses or beans
		Percentage of women consuming fruits
Gender roles	Gender role attitudes	Percentage of women who agree that a husband is justified in hitting or beating his wife for at least one specified reason
		Percentage of women who agree that a wife is justified in refusing to have sex with her husband for all specified reasons
		Percentage of women who agree that when a wife refuses to have sex with her husband, he does not have the right to any of the four specified behaviors
	Violence	Physical violence
		Sexual violence
		Violence during pregnancy
		Emotional violence

The correlates chosen for this study were based on existing scientific evidence of their impact on women’s empowerment. These included the per capita net state domestic product (current prices), the percentage of institutional deliveries, female suicide rates, maternal mortality rates, educational indicators like the percentage of literate women, those with more than ten years of schooling, the median age at marriage for women, and overall fertility rates [11-13].

Statistical analysis

Data analysis was done using MS Excel version 2016 (Microsoft Corporation, Redmond, Washington, United States) and Stata version 18 (StataCorp LLC, College Station, Texas, United States). The WEI was calculated for each of the 28 states. To check for the normality of the data, the Shapiro-Wilk test was done. Pairwise correlation was performed to assess the association of independent variables with the dependent variable. Pearson correlation was done for normally distributed data, and for non-normal data, Spearman correlation was done. The reported value of Pearson’s r was interpreted to provide an understanding of the strength and direction of the relationship between the two variables. The p-value was set at 0.05 for significance.

Ethical approval

This study is limited to publicly available data collected as part of routine periodic surveillance. No individual data records were used in this study. Access to the NFHS-5 data for the current research purpose was authorized by the DHS.

## Results

**Figure 2:**
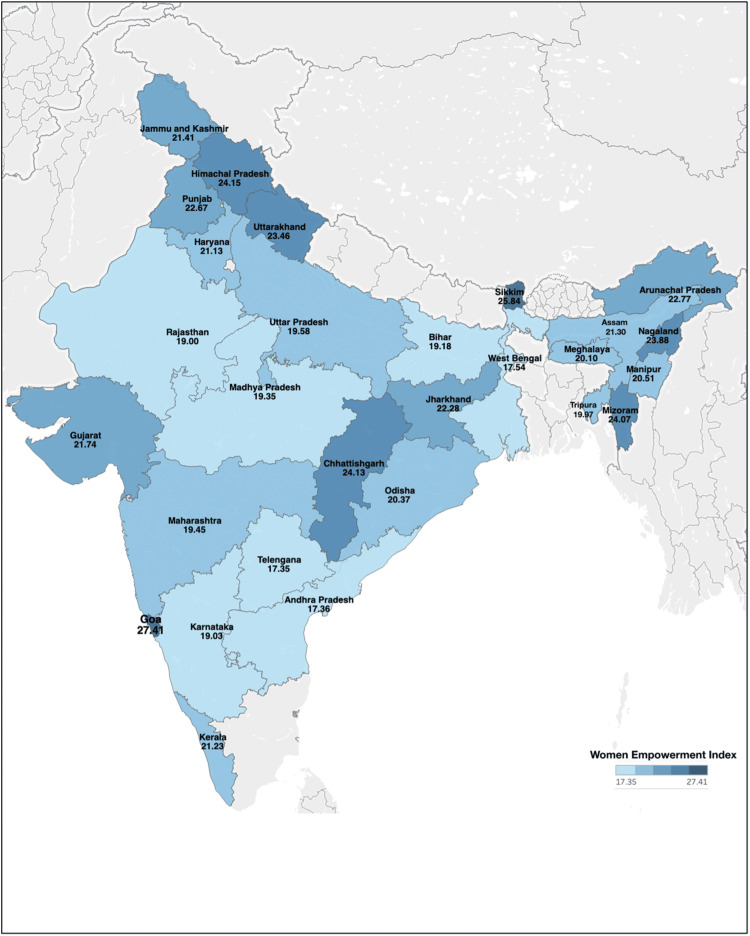
WEI across 28 states of India WEI, Women’s Empowerment Index

The WEI was calculated for each state using a combination of economic, social, and health-related indicators. The analysis focuses on the variations in WEI and its subindices, as well as the correlations between WEI and various socioeconomic variables. The WEI ranged from 17.4 to 27.4 among the 28 states included in the study. The mean WEI was found to be 21.3 ± 2.6. The states of Goa (27.4), Sikkim (25.9), and Himachal Pradesh (24.2) exhibited the highest levels of women empowerment, while West Bengal (17.5), Andhra Pradesh (17.4), and Telangana (17.4) showed the lowest levels (Table 2, Figure 2).

**Table 2:** Levels of WEI and its four subindices across 28 states of India WEI, Women’s Empowerment Index

State	Economic empowerment (0-9)	Decision-making (0-6)	Health and nutrition (0-17)	Gender roles (0-7)	WEI (0-39)
Andhra Pradesh	4.5	1.24	8.47	3.14	17.36
Arunachal Pradesh	6.05	2.73	10.37	3.61	22.77
Assam	3.04	3.21	10.97	4.07	21.3
Bihar	3.6	2.86	9.21	3.52	19.18
Chhattisgarh	4.53	3.91	10.01	5.68	24.13
Goa	4.93	3.98	12.42	6.08	27.41
Gujarat	3.87	3.77	8.51	5.59	21.74
Haryana	3.53	2.66	9.49	5.45	21.13
Himachal Pradesh	4.4	2.87	10.26	6.62	24.15
Jammu and Kashmir	5.11	0.8	10.9	4.59	21.41
Jharkhand	4.67	3.47	9.6	4.55	22.28
Karnataka	6.41	1	10.65	0.97	19.03
Kerala	3.28	2.49	10.14	5.32	21.23
Madhya Pradesh	3.32	2.16	9.4	4.47	19.35
Maharashtra	3.57	2.43	9.11	4.34	19.45
Manipur	3.97	3.57	9.55	3.42	20.51
Meghalaya	4.55	3.54	7.93	4.08	20.1
Mizoram	3.48	4.5	10.69	5.4	24.07
Nagaland	2.69	5.64	9.28	6.27	23.88
Odisha	4.25	2.53	9.31	4.28	20.37
Punjab	4.78	3.66	9.11	5.11	22.67
Rajasthan	2.95	1.75	10.16	4.15	19
Sikkim	6.1	2.92	11.85	4.98	25.84
Telangana	5.32	1.08	8.48	2.47	17.35
Tripura	3.21	3.49	8.58	4.7	19.97
Uttar Pradesh	3.33	2.58	9.17	3.98	19.06
Uttarakhand	3.3	3.03	11.2	5.93	23.46
West Bengal	3.11	2.57	8.99	2.87	17.54

**Figure 3:**
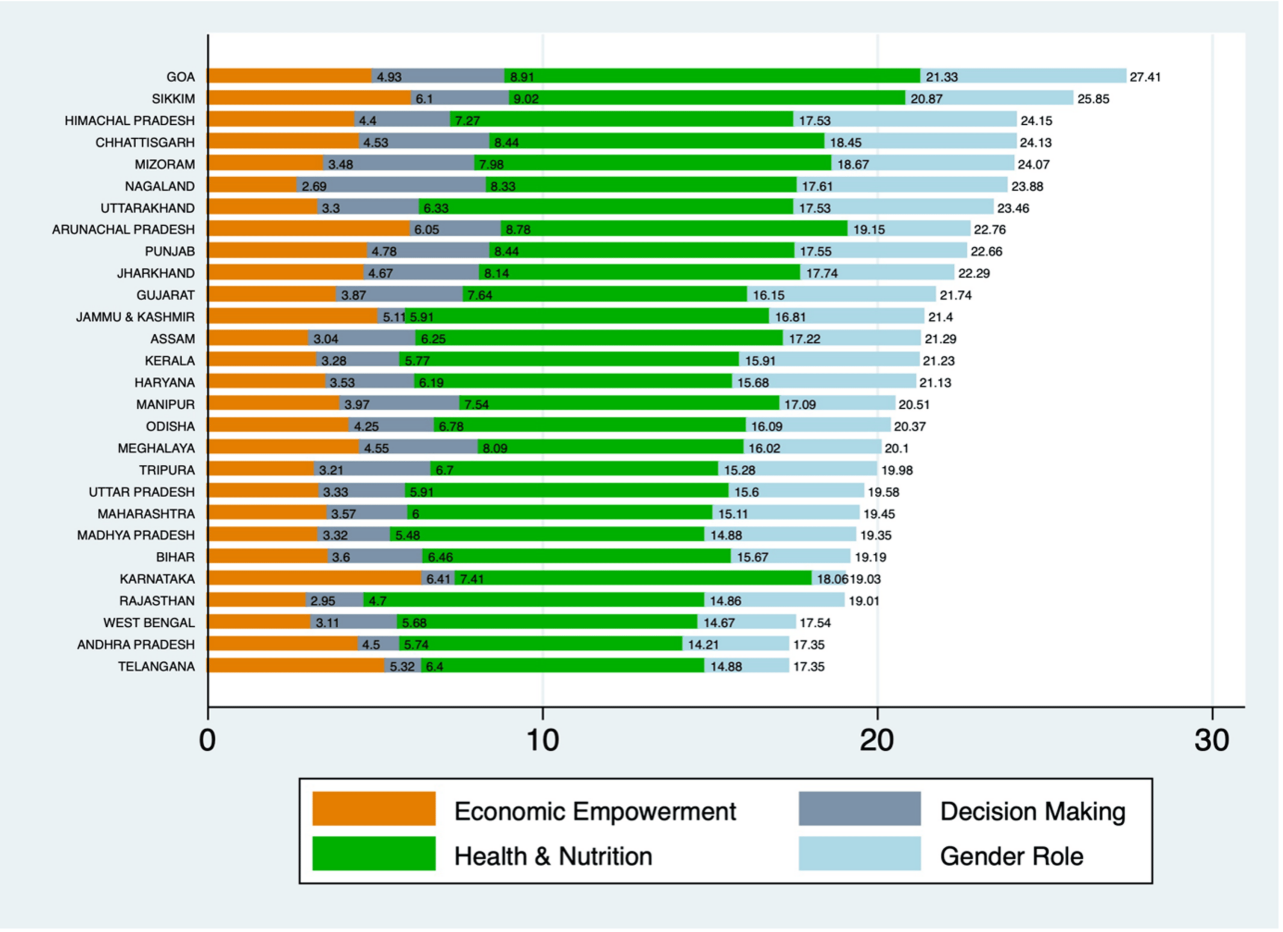
WEI and subindices by state WEI, Women’s Empowerment Index

Economic empowerment scores ranged from 2.7 in Nagaland to 6.4 in Karnataka. Karnataka (6.4), Sikkim (6.1), and Arunachal Pradesh (6.1) had the highest scores, indicating better economic opportunities and resources for women. In contrast, Assam (3.1), Rajasthan (2.9), and Nagaland (2.7) had the lowest scores. The decision-making subindex varied significantly among states, with Nagaland (5.6), Mizoram (4.5), and Goa (3.9) scoring the highest. Telangana (1.1), Karnataka (1.0), and Jammu and Kashmir (0.8) had the lowest scores, indicating limited participation of women in household decision-making processes. Health and nutrition scores were highest in Goa (12.4), Sikkim (11.9), and Uttarakhand (11.2), reflecting better health outcomes and nutritional status. Meghalaya (7.9), Telangana (8.5), and Andhra Pradesh (8.5) were among the states with lower scores in this dimension. Positive gender roles were most prominent in Himachal Pradesh (6.6), Nagaland (6.3), and Goa (6.8). West Bengal (2.9), Telangana (2.5), and Karnataka (0.9) showed the least empowerment in terms of gender role attitudes (Figure 3).

Spearman and Pearson correlations were performed to assess the association between WEI and various socioeconomic variables. Variables such as per capita net state domestic product, percentage of literate women, percentage of women with more than ten years of schooling, and women's median age at marriage were significantly and positively correlated with WEI. The total fertility rate was significantly and negatively correlated with WEI (Table 3).

**Table 3:** Pairwise correlations between WEI and other variables * Statistically significant (p-value <0.05) WEI, Women’s Empowerment Index

S. no.	Variable	Correlation	p-value
1	Per capita net state domestic product (current prices)	0.515	<0.01*
2	Female suicide rates per 1 lakh female population	-0.236	0.226
3	Percentage of institutional deliveries	0.001	0.997
4	Percentage of literate women	0.488	<0.01*
5	Percentage of women who completed more than 10 years of schooling	0.428	0.023*
6	Infant mortality rate	-0.364	0.057
7	Maternal mortality rate	-0.047	0.811
8	Total fertility rate	-0.411	0.030*
9	Median age at marriage	0.562	<0.01*

## Discussion

The present study highlights significant variations in women’s empowerment across different states in India, as measured by the WEI. The results underscore the importance of socioeconomic, health, and educational factors in shaping women’s empowerment. The WEI revealed substantial disparities across the 28 states studied, with Goa, Sikkim, and Himachal Pradesh exhibiting the highest levels of women’s empowerment, while West Bengal, Andhra Pradesh, and Telangana had the lowest. These variations can be attributed to differences in socioeconomic development, cultural norms, and state-specific policies.

Economic empowerment scores varied significantly, with Karnataka, Sikkim, and Arunachal Pradesh scoring the highest. These states benefit from state-led initiatives that improve women’s access to employment and credit, highlighting that economic opportunities and access to resources are crucial for women’s empowerment. Studies have shown that financial independence enhances women's decision-making power and overall well-being [14]. Conversely, states with lower economic empowerment scores, such as Assam and Rajasthan, often face challenges like limited job opportunities and sociocultural barriers that restrict women’s participation in the workforce. Addressing these barriers is essential for improving women's economic empowerment in these regions.

States leading the way in women's engagement in home and societal decision-making include Nagaland, Mizoram, and Goa, according to the subindex on decision-making. In these states, community-driven strategies and a generally equal social framework have made it easier for women to participate at higher levels in decision-making [2]. In contrast, states like Telangana and Jammu and Kashmir, which scored low in this subindex, reflect a need for targeted interventions to enhance women’s decision-making capabilities. Programs that promote gender equality within households and communities could be effective in these regions.

Health and nutrition scores were highest in Goa, Sikkim, and Uttarakhand, reflecting better health outcomes and nutritional status for women. Access to quality healthcare and nutritional support is fundamental for women’s empowerment [15]. These states have benefited from robust healthcare infrastructure and targeted health programs. States with lower health and nutrition scores, such as Meghalaya and Telangana, need to improve healthcare access and address nutritional deficiencies. Investing in maternal and child health programs and ensuring that women have access to essential health services can significantly enhance empowerment in these areas.

Positive gender roles were most prominent in Himachal Pradesh, Nagaland, and Goa, where significant strides have been made in challenging traditional gender norms through public awareness campaigns and gender-sensitive policies [1]. In contrast, states with lower scores in this subindex, such as West Bengal and Telangana, must intensify efforts to dismantle patriarchal norms and promote gender equality. Education and advocacy are vital tools for changing societal attitudes toward gender roles.

The correlation analysis revealed that per capita net state domestic product, literacy rates, and median age at marriage were positively correlated with WEI, while total fertility rate was negatively correlated. These findings align with existing literature, which suggests that economic development, education, and delayed marriage contribute to women’s empowerment [16]. A higher per capita income allows for better access to resources and opportunities, which in turn enhances empowerment. Delaying marriage gives women more time to pursue their jobs and education while also providing them with the knowledge and skills necessary for both personal and professional success [2].

Incorporating findings from recent studies on women’s empowerment in India, several additional barriers and strategies have been identified. Economic barriers, such as limited job opportunities and financial dependence, significantly impact women’s empowerment. Sociocultural barriers and a lack of job opportunities, particularly in rural areas, hinder economic independence [14,15]. Financial dependence on male family members restricts women’s decision-making power [17].

Educational barriers also play a crucial role, with significant gender gaps in literacy rates and many women receiving little to no education. Societal pressures, child marriages, and a lack of facilities contribute to high dropout rates among girls [18,19]. Health barriers, such as lack of access to quality healthcare services in rural areas, affect women’s overall well-being and participation in the workforce [20]. High maternal mortality rates indicate poor maternal health services [21].

Cultural and social barriers, including patriarchal norms and traditional gender roles, restrict women’s autonomy and freedom [22,23]. Persistent gender biases and discriminatory practices limit access to resources, education, and employment [24,25]. Political barriers, such as the underrepresentation of women in political positions, limit their influence on policy-making and governance.

To improve women’s empowerment in India, several strategies can be implemented. Economic empowerment can be achieved by creating job opportunities and promoting entrepreneurship through microfinance and financial support programs. Enhancing financial inclusion initiatives to provide women with access to credit, savings, and other financial services is also crucial [17]. Educational empowerment requires ensuring equal access to quality education for girls at all levels and implementing programs to reduce school dropout rates [19]. Providing scholarships and financial incentives to encourage higher education among women is essential. Healthcare improvement involves improving access to maternal and reproductive health services [20]. Implementing health awareness programs to educate women about their health rights and available services is also important [21]. Cultural and social changes can be facilitated through public awareness campaigns to challenge traditional gender norms and stereotypes [1]. Developing community programs that promote gender equality and empower women to take on leadership roles within their communities is vital [22]. Political empowerment can be achieved by promoting greater representation of women in political positions through affirmative action and gender quotas [25]. Advocating for policy reforms that address gender inequalities and support women’s rights and empowerment is necessary [23,26].

This study is pivotal as it delivers a thorough analysis of women’s empowerment across various states in India, uncovering the disparities and the reasons behind them. By pinpointing specific areas requiring improvement, it provides valuable insights for policymakers and stakeholders committed to promoting gender equality and empowering women. However, the study mainly focuses on certain correlates of women’s empowerment, potentially overlooking other crucial factors such as political stability, environmental conditions, and technological advancements that could also play a role. Additionally, while correlations between various factors and women’s empowerment are identified, the study does not establish causal relationships, necessitating further research to determine these dynamics. Continuous assessment and strategy adaptation will be essential in making sustained progress toward achieving gender equality and empowering women nationwide.

## Conclusions

This study reveals significant state-wise variations in women’s empowerment across India, with Goa, Sikkim, and Himachal Pradesh showing the highest levels, while West Bengal, Andhra Pradesh, and Telangana exhibit the lowest. Economic opportunities, educational access, healthcare quality, and positive gender roles are critical for enhancing empowerment. The positive correlations between WEI and socioeconomic variables like per capita income, literacy rates, and median age at marriage, and the negative correlation with total fertility rate, underscore the multifaceted nature of women’s empowerment. Targeted interventions addressing these factors are essential for promoting gender equality and empowering women across India.
